# Equivalent conditions of complete moment convergence for extended negatively dependent random variables

**DOI:** 10.1186/s13660-017-1403-2

**Published:** 2017-05-31

**Authors:** Qunying Wu, Xiang Zeng

**Affiliations:** College of Science, Guilin University of Technology, Guilin, 541004 P.R. China

**Keywords:** 60F15, extended negatively dependent random variables, complete moment convergence, moment condition, equivalent conditions

## Abstract

In this paper, we study the equivalent conditions of complete moment convergence for sequences of identically distributed extended negatively dependent random variables. As a result, we extend and generalize some results of complete moment convergence obtained by Chow (Bull. Inst. Math. Acad. Sin. 16:177-201, 1988) and Li and Spătaru (J. Theor. Probab. 18:933-947, 2005) from the i.i.d. case to extended negatively dependent sequences.

## Introduction

Random variables *X* and *Y* are said to be negative quadrant dependent (NQD) if

1.1 $$ P(X\leq x,Y\leq y)\leq P(X\leq x)P(Y\leq y) $$

for all $x,y\in\mathrm{R}$. A collection of random variables is said to be pairwise negative quadrant dependent (PNQD) if every pair of random variables in the collection satisfies (1.1).

It is important to note that (1.1) implies

1.2 $$ P(X> x,Y> y)\leq P(X> x)P(Y> y) $$

for all $x,y\in\mathrm{R}$. Moreover, it follows that (1.2) implies (1.1), and hence (1.1) and (1.2) are equivalent. However, Ebrahimi and Ghosh (1981 [3]) showed that (1.1) and (1.2) are not equivalent for a collection of three or more random variables. Accordingly, the following definition is needed to define sequences of extended negatively dependent random variables.

### Definition 1.1

Random variables $X_{1},\ldots,X_{n}$ are said to be extended negatively dependent (END) if there exists a constant $M>0$ such that for all real $x_{1},\ldots,x_{n}$,

$$\begin{aligned}& P \Biggl(\bigcap_{j=1}^{n}(X_{j} \leq x_{j}) \Biggr)\leq M \prod_{j=1}^{n}P(X_{j} \leq x_{j}), \\& P \Biggl(\bigcap_{j=1}^{n}(X_{j} > x_{j}) \Biggr)\leq M \prod_{j=1}^{n}P(X_{j}> x_{j}). \end{aligned}$$

An infinite sequence of random variables $\{X_{n};n\geq1\}$ is said to be END if every finite subset $X_{1},\ldots,X_{n}$ is END.

### Definition 1.2

Random variables $X_{1},X_{2},\ldots,X_{n}$, $n\geq2$, are said to be negatively associated (NA) if for every pair of disjoint subsets $A_{1}$ and $A_{2}$ of $\{1,2,\ldots,n\}$,

$$\operatorname{cov}\bigl(f_{1}(X_{i}; i\in A_{1}),f_{2}(X_{j}; j\in A_{2})\bigr) \leq0, $$

where $f_{1}$ and $f_{2}$ are increasing for every variable (or decreasing for every variable) functions such that this covariance exists. A sequence of random variables $\{X_{i}; i\geq1\}$ is said to be NA if its every finite subfamily is NA.

The definition of PNQD was given by Lehmann (1966 [4]). The definition of NA was introduced by Joag-Dev and Proschan (1983 [5]), and the concept of END was given by Liu (2009 [6]). In the case $M=1$, the notion of END random variables reduces to the well-known notion of the so-called negatively dependent (ND) random variables which was introduced by Bozorgnia et al. (1993 [7]). These concepts of dependent random variables are very useful in reliability theory and applications.

It is easy to see from the definitions that NA implies ND and END. But example 1.5 in Wu and Jiang (2011 [8]) shows that ND or END does not imply NA. Thus, it is shown that END is much weaker than NA. In the articles listed earlier, a number of well-known multivariate distributions are shown to possess the END properties. In many statistical and mechanic models, an END assumption among the random variables in the models is more reasonable than an independent or NA assumption. Because of wide applications in multivariate statistical analysis and reliability theory, the notions of END random variables have attracted more and more attention recently. A series of useful results have been established (cf. Liu (2009 [6]), Chen et al. (2010 [9]), Shen (2011 [10], 2016 [11]), Wu et al. (2014 [12]), Liu et al. (2015 [13]), Qiu and Chen (2015 [14]), Wang et al. (2015 [15]), Xu et al. (2016 [16]), and Wang and Hu (2017 [17])). Hence, it is highly desirable and of considerable significance in the theory and application to study the limit properties of END random variables theorems and applications.

Chow (1988 [1]) first investigated the complete moment convergence, which is more exact than complete convergence. Thus, complete moment convergence is one of the most important problems in probability theory. Recent results can be found in Chen and Wang (2008 [18]), Gut and Stadtmller (2011 [19]), Sung (2013 [20]), Wang and Hu (2014 [21]), Guo (2014 [22]), Qiu (2014 [23]), Qiu and Chen (2014 [24]), Wu and Jiang (2016 [25]) and Wu and Jiang (2016 [26]). In addition, Li and Spătaru (2005 [2]) obtained the following complete moment convergence theorem: Let $\{X,X_{n};n\geq1\}$ be a sequence of independent and identically distributed (i.i.d.) random variables with partial sums $S_{n}=\sum_{i=1}^{n}X_{i}$, $n\geq1$. Suppose that $r\geq1$, $0< p<2$, $q>0$. Then

$$ \textstyle\begin{cases} \mathbb{E}|X|^{rp} < \infty,&\mbox{if }q< rp, \\ \mathbb{E}|X|^{rp} \ln|X|< \infty,&\mbox{if }q=rp, \\ \mathbb{E}|X|^{q} < \infty,&\mbox{if }q>rp, \end{cases} $$

if and only if

1.3 $$ \int_{\varepsilon}^{\infty}\sum_{n=1}^{\infty}n^{r-2}P \bigl(|S_{n}-nb|>x^{1/q} n^{1/p} \bigr)\, \mathrm{d}x< \infty \quad \mbox{for any } \varepsilon>0, $$

where $b=\mathbb{E}X$ if $rp\geq1$ and $b=0$ if $0< rp<1$.

Furthermore, Chen and Wang (2008 [18]) showed that (1.3) and

$$ \sum_{n=1}^{\infty}n^{r-2-q/p}\mathbb{E} \bigl\{ \vert S_{n}-nb \vert -\varepsilon \bigr\} _{+}^{q}< \infty\quad \mbox{for any } \varepsilon>0 $$

are equivalent.

## Conclusions

The purpose of this paper is to study and establish the equivalent conditions of complete moment convergence of the maximum of the absolute value of the partial sum $\max_{1\leq k\leq n}|S_{k}|$ for sequences of identically distributed extended negatively dependent random variables. Our results not only extend and generalize some results on the complete moment convergence such as obtained by Chow (1988 [1]) and Li and Spătaru (2005 [2]) from the i.i.d. case to extended negatively dependent sequences, but also from partial sums case to the maximum of partial sums. Our research results and research methods provide some useful ideas and methods for the study of the complete moment convergence of the maximum of partial sums for other dependent random variables.

In the following, the symbol *c* stands for a generic positive constant which may differ from one place to another. Let $a_{n}\ll b_{n}$ denote that there exists a constant $c>0$ such that $a_{n}\leq cb_{n}$ for sufficiently large *n*, ln*x* means $\ln(\max(x,\mathrm{e}))$ and *I* denotes an indicator function.

### Theorem 2.1

*Let* $\{X,X_{n};n\geq1\}$ *be a sequence of identically distributed END random variables with partial sums* $S_{n}=\sum_{i=1}^{n}X_{i}$, $n\geq1$. *Suppose that* $r>1$, $0< p<2$, $q>0$ *and* $\mathbb{E}X=0$ *for* $1\leq p<2$. *Then the following statements are equivalent*:

2.1 $$\begin{aligned}& \textstyle\begin{cases}\mathbb{E}|X|^{rp} < \infty,&\textit{if }q< rp, \\ \mathbb{E}|X|^{rp} \ln|X|< \infty,&\textit{if } q=rp, \\ \mathbb{E}|X|^{q} < \infty,&\textit{if }q>rp, \end{cases}\displaystyle \end{aligned}$$

2.2 $$\begin{aligned}& \sum_{n=1}^{\infty}n^{r-2-q/p}\mathbb{E} \Bigl\{ \max_{1\leq k\leq n}|S_{k}|-\varepsilon n^{1/p} \Bigr\} _{+}^{q}< \infty \quad \textit{for any } \varepsilon>0, \end{aligned}$$

2.3 $$\begin{aligned}& \sum_{n=1}^{\infty}n^{r-2}\mathbb{E} \Bigl\{ \sup_{k\geq n}k^{-1/p}|S_{k}|-\varepsilon \Bigr\} _{+}^{q}< \infty\quad \textit{for any } \varepsilon>0. \end{aligned}$$

### Remark 2.2

Our Theorem 2.1 not only generalizes the corresponding results obtained by Chow (1988 [1]) and Li and Spătaru (2005 [2]) from the i.i.d. case to END sequences, but also $|S_{n}|$ being replaced by $\max_{1\leq k\leq n}|S_{k}|$. So Theorem 2.1 generalizes and improves the corresponding results obtained by Chow (1988 [1]) and Li and Spătaru (2005 [2]).

## Proofs

The following three lemmas play important roles in the proof of our theorems. Lemma 3.1 can be obtained directly from the definition of END sequences.

### Lemma 3.1

*Let* $\{X_{n}; n\geq1\}$ *be a sequence of END random variables and* $\{f_{n}; n\geq1\}$ *be a sequence of Borel functions*, *all of which are monotone increasing* (*or all are monotone decreasing*). *Then* $\{f_{n}(X_{n}); n\geq1\}$ *is a sequence of END r*.*v*.*’s*.

### Lemma 3.2

Liu et al. 2015 [13]

*Let* $\{ X_{n};n\geq1\}$ *be a sequence of END random variables with* $\mathbb{E}X_{n}=0$ *and* $\mathbb {E}|X_{n}|^{p}<\infty$, $p\geq 2$. *Then there exists a positive constant* *c* *depending only on* *p* *such that*

$$\mathbb{E}\bigl(|S_{n}|^{p}\bigr)\leq c \Biggl\{ \sum ^{n} _{i=1}\mathbb{E}|X_{i}|^{p} + \Biggl(\sum^{n} _{i=1} \mathbb{E}X_{i}^{2} \Biggr)^{p/2} \Biggr\} $$

*and*

$$\mathbb{E}\Bigl(\max_{1\leq i \leq n}|S_{i}|^{p} \Bigr)\leq c\ln^{p}n \Biggl\{ \sum^{n} _{i=1}\mathbb{E}|X_{i}|^{p} + \Biggl(\sum ^{n} _{i=1}\mathbb{E}X_{i}^{2} \Biggr)^{p/2} \Biggr\} . $$

### Lemma 3.3

*Let* $\{X_{n};n\geq1\}$ *be a sequence of END random variables*. *Then*, *for any* $x\geq0$, *there exists a positive constant* *c* *such that for all* $n\geq1$,

$$\Bigl(1-P\Bigl(\max_{1\leq k\leq n} \vert X_{k} \vert >x \Bigr) \Bigr)^{2}\sum_{k=1} ^{n}P \bigl( \vert X_{k} \vert >x\bigr)\leq cP \Bigl(\max _{1\leq k\leq n} \vert X_{k} \vert >x \Bigr). $$

Further, if $P(\max_{1\leq k\leq n}|X_{k}|>x)\rightarrow0$ as $n\rightarrow\infty$, then there exists a positive constant *c* such that for all $n\geq1$,

$$\sum_{k=1} ^{n}P\bigl( \vert X_{k} \vert >x\bigr)\leq cP \Bigl(\max_{1\leq k\leq n} \vert X_{k} \vert >x \Bigr). $$

### Proof

From the proof of Lemma 1.4 in Wu (2012 [27]) and by Lemma 3.2, we can prove Lemma 3.3. □

### Proof of Theorem 2.1

We first prove that (2.1) ⇒ (2.2). Note that

$$\begin{aligned}& \sum_{n=1}^{\infty}n^{r-2-q/p}\mathbb{E} \Bigl\{ \max_{1\leq k\leq n}|S_{k}|-\varepsilon n^{1/p} \Bigr\} _{+}^{q} \\& \quad = \sum_{n=1}^{\infty}n^{r-2-q/p} \int_{0}^{n^{1/p}} q x^{q-1} P \Bigl(\max _{1\leq k\leq n}|S_{k}|-\varepsilon n^{1/p}>x \Bigr)\, \mathrm{d}x \\& \qquad {}+\sum_{n=1}^{\infty}n^{r-2-q/p} \int_{n^{1/p}}^{\infty} q x^{q-1}P \Bigl(\max _{1\leq k\leq n}|S_{k}|-\varepsilon n^{1/p}>x \Bigr)\, \mathrm{d}x \\& \quad \ll \sum_{n=1}^{\infty}n^{r-2}P \Bigl(\max_{1\leq k\leq n}|S_{k}|>\varepsilon n^{1/p} \Bigr) \\& \qquad {}+\sum_{n=1}^{\infty}n^{r-2-q/p} \int_{n^{1/p}}^{\infty} x^{q-1}P \Bigl(\max _{1\leq k\leq n}|S_{k}|>x \Bigr)\,\mathrm{d}x \\& \quad \mathop{\hat{=}} I+J. \end{aligned}$$

By Corollary 2.1 in Liu et al. (2015 [13]), $I<\infty$. Hence, in order to establish (2.2), it is enough to prove that

3.1 $$ J\mathop{\hat{=}}\sum_{n=1}^{\infty}n^{r-2-q/p} \int_{n^{1/p}}^{\infty} x^{q-1}P \Bigl(\max _{1\leq k\leq n}|S_{k}|>x \Bigr)\,\mathrm{d}x< \infty. $$

Let $x\geq n^{1/p}$, $r^{-1}<\alpha<1$ and an integer $N>\max ((r-1)(\alpha r-1)^{-1}, q(\alpha rp)^{-1})$. Define, for $1\leq k\leq n$, $n\geq1$,

$$\begin{aligned}& X_{k}^{(1)}=-x^{\alpha}I\bigl(X_{k}< -x^{\alpha}\bigr)+X_{k}I\bigl(|X_{k}|\leq x^{\alpha}\bigr)+x^{\alpha}I\bigl(X_{k}>x^{\alpha}\bigr), \\& X_{k}^{(2)}=\bigl(X_{k}-x^{\alpha}\bigr) I \bigl(x^{\alpha}< X_{k}< x/(4N)\bigr), \\& X_{k}^{(3)}=\bigl(X_{k}+x^{\alpha}\bigr) I \bigl(-x/(4N)< X_{k}< -x^{\alpha}\bigr), \\& X_{k}^{(4)}=\bigl(X_{k}+x^{\alpha}\bigr) I \bigl(X_{k}\leq-x/(4N)\bigr)+\bigl(X_{k}-x^{\alpha}\bigr) I\bigl(X_{k}\geq x/(4N)\bigr), \\& S^{(j)}_{k}=\sum_{i=1}^{k}X^{(j)}_{i}, \quad j=1, 2, 3, 4. \end{aligned}$$

It is obvious that $S_{k}=\sum_{j=1}^{4}S^{(j)}_{k}$. Hence, in order to establish (3.1), it suffices to prove that

3.2 $$ J^{(j)}\mathop{\hat{=}}\sum_{n=1}^{\infty}n^{r-2-q/p} \int_{n^{1/p}}^{\infty} x^{q-1}P \Bigl(\max _{1\leq k\leq n} \bigl\vert S^{(j)}_{k} \bigr\vert >x/4 \Bigr)\,\mathrm{d}x< \infty, \quad j=1, 2, 3, 4. $$

Note that

$$ \sum_{n=1}^{j}n^{r-1-q/p}\ll \textstyle\begin{cases} j^{r-q/p},&\mbox{if }q< rp, \\ \ln j,&\mbox{if }q=rp, \\ 1,&\mbox{if }q>rp. \end{cases} $$

By combining this with (2.1), Markov’s inequality and

$$\begin{aligned} P\Bigl(\max_{1\leq k\leq n} \bigl\vert S^{(4)}_{k} \bigr\vert >x/4\Bigr) \leq& P\bigl(\mbox{there is } k, k\in[1, n] \mbox{ such that } X^{(4)}_{k}\neq0\bigr) \\ \leq& nP\bigl( \vert X \vert \geq x/(4N)\bigr), \end{aligned}$$

we get

3.3 $$\begin{aligned} J^{(4)} \leq& \sum_{n=1}^{\infty}n^{r-1-q/p} \int_{n^{1/p}}^{\infty} x^{q-1}P\bigl( \vert X \vert >x/(4N)\bigr)\,\mathrm{d}x \\ \leq&\sum_{n=1}^{\infty}n^{r-1-q/p}\sum _{j=n}^{\infty}\int _{j^{1/p}}^{(j+1)^{1/p}} x^{q-1}P\bigl(4N \vert X \vert >j^{1/p}\bigr)\,\mathrm{d}x \\ \ll&\sum_{n=1}^{\infty}n^{r-1-q/p}\sum _{j=n}^{\infty}j^{q/p-1}P\bigl(4N \vert X \vert >j^{1/p}\bigr) \\ =&\sum_{j=1}^{\infty}\sum _{n=1}^{j} n^{r-1-q/p} j^{q/p-1}P\bigl(4N \vert X \vert >j^{1/p}\bigr) \\ \ll& \textstyle\begin{cases} \sum_{j=1}^{\infty}j^{r-1}P(4N \vert X \vert >j^{1/p}),&\mbox{if }q< rp, \\ \sum_{j=1}^{\infty}j^{r-1}\ln j P(4N \vert X \vert >j^{1/p}),&\mbox{if }q=rp, \\ \sum_{j=1}^{\infty}j^{q/p-1}P(4N \vert X \vert >j^{1/p}),&\mbox{if }q>r p \end{cases}\displaystyle \\ \ll& \textstyle\begin{cases} \mathbb{E} \vert X \vert ^{rp} < \infty,&\mbox{if }q< rp, \\ \mathbb{E} \vert X \vert ^{rp} \ln \vert X \vert < \infty,&\mbox{if }q=rp, \\ \mathbb{E} \vert X \vert ^{q} < \infty,&\mbox{if }q>rp. \end{cases}\displaystyle \end{aligned}$$

From the definition of $X^{(2)}_{k}$, it is clear that $X^{(2)}_{k}>0$. Thus, by Definition 1.1 and (2.1),

$$\begin{aligned} P \Bigl(\max_{1\leq k\leq n} \bigl\vert S^{(2)}_{k} \bigr\vert >x/4 \Bigr) =&P \Biggl(\sum_{k=1}^{n}X^{(2)}_{k}>x/4 \Biggr) \\ \leq& P\bigl(\mbox{there are at least } N \mbox{ indices } i\in[1, n] \mbox{ such that } X_{i}>x^{\alpha}\bigr) \\ \leq&\sum_{1\leq i_{1}< i_{2}< \cdots< i_{N}\leq n}\prod_{j=1}^{N}P \bigl( \vert X_{i_{j}} \vert >x^{\alpha}\bigr) \\ \leq& n^{N}\bigl(P\bigl( \vert X \vert >x^{\alpha}\bigr) \bigr)^{N}\leq n^{N}x^{-\alpha rpN}\bigl(\mathbb{E} \vert X \vert ^{rp}\bigr)^{N} \\ \ll& n^{N}x^{-\alpha rpN}. \end{aligned}$$

Hence, by the definition of *N*, $-\alpha rpN+q-1<-1$ and $-(\alpha r-1)N+r-2<-1$,

3.4 $$\begin{aligned} J^{(2)} \ll&\sum_{n=1}^{\infty}n^{r-2-q/p+N} \int_{n^{1/p}}^{\infty} x^{-\alpha rpN +q-1}\,\mathrm{d}x \\ \ll& \sum_{n=1}^{\infty}n^{r-2-q/p+N}n^{(-\alpha rpN +q)/p} \\ =&\sum_{n=1}^{\infty}n^{-(\alpha r-1)N-2+r} \\ < &\infty. \end{aligned}$$

Similarly, we can show

3.5 $$ J^{(3)}< \infty. $$

In order to estimate $J^{(1)}$, we first verify that

3.6 $$ \sup_{x\geq n^{1/p}}\max_{1\leq k\leq n}x^{-1}\bigl| \mathbb{E}S^{(1)}_{k}\bigr|\rightarrow0\quad \mbox{as } n \rightarrow\infty. $$

When $0< p<1$, by Markov’s inequality and $\mathbb{E}|X|^{p}<\infty$,

$$\begin{aligned} \sup_{x\geq n^{1/p}}\max_{1\leq k\leq n}x^{-1} \bigl\vert \mathbb{E}S^{(1)}_{k} \bigr\vert \leq&\sup _{x\geq n^{1/p}}x^{-1}n \bigl(x^{\alpha}P\bigl( \vert X \vert >x^{\alpha}\bigr)+\mathbb{E} \vert X \vert I\bigl( \vert X \vert \leq x^{\alpha}\bigr) \bigr) \\ \leq&\sup_{x\geq n^{1/p}} \bigl(nx^{-1+\alpha-\alpha p} \mathbb{E} \vert X \vert ^{p}+x^{-1}n\mathbb{E} \vert X \vert ^{p}x^{\alpha(1-p)} \bigr) \\ \ll& n^{-(1-\alpha)(p^{-1}-1)} \\ \rightarrow&0\quad \mbox{as } n\rightarrow\infty. \end{aligned}$$

When $1\leq p<2$, by $\mathbb{E}X=0$ and $\mathbb{E}|X|^{rp}<\infty$, we get

$$\begin{aligned} \sup_{x\geq n^{1/p}}\max_{1\leq k\leq n}x^{-1} \bigl\vert \mathbb{E}S^{(1)}_{k} \bigr\vert \leq&\sup _{x\geq n^{1/p}} \bigl(n x^{-1+\alpha}P\bigl( \vert X \vert >x^{\alpha}\bigr)+x^{-1}n\mathbb {E} \vert X \vert I\bigl( \vert X \vert > x^{\alpha}\bigr) \bigr) \\ \ll&\sup_{x\geq n^{1/p}}nx^{-1-\alpha rp+\alpha}\mathbb{E} \vert X \vert ^{rp}\ll n^{-(\alpha r-1)-(1-\alpha)/p} \\ \rightarrow&0 \quad \mbox{as } n\rightarrow\infty. \end{aligned}$$

That is, (3.6) holds. Hence, in order to prove $J^{(1)}<\infty$, it suffices to prove that

3.7 $$ \tilde{J}^{(1)} \mathop{\hat{=}} \sum_{n=1}^{\infty}n^{r-2-q/p} \int_{n^{1/p}}^{\infty} x^{q-1}P \Bigl(\max _{1\leq k\leq n} \bigl\vert S^{(1)}_{k}- \mathbb{E}S^{(1)}_{k} \bigr\vert >x/8 \Bigr)\,\mathrm{d}x< \infty. $$

Obviously, $X^{(1)}_{k}$ is increasing on $X_{k}$, thus by Lemma 3.1, $\{X^{(1)}_{k}; k\geq1\}$ is also a sequence of END random variables. In view of Lemma 3.2, taking $u=\max(rp,q)$ and

$$s>\max \biggl(2, u, \frac{r-1}{\frac{1}{2} (\frac{\alpha u}{p}-1 )+\frac{1-\alpha}{p}}, \frac{r-1}{\frac{1}{p}-\frac{1}{2}}, \frac{q}{1-\alpha+\frac{\alpha u}{2}}, \frac{q-\alpha rp}{1-\alpha} \biggr), $$

we obtain

3.8 $$\begin{aligned} \tilde{J}^{(1)} \ll& \sum_{n=1}^{\infty}n^{r-2-q/p} \int_{n^{1/p}}^{\infty} x^{q-1-s}\mathbb{E} \Bigl(\max _{1\leq k\leq n} \bigl\vert S^{(1)}_{k}- \mathbb{E}S^{(1)}_{k} \bigr\vert ^{s} \Bigr)\, \mathrm{d}x \\ \ll& \sum_{n=1}^{\infty}n^{r-1-q/p} \ln^{s} n \int_{n^{1/p}}^{\infty} x^{q-1-s}\mathbb{E} \bigl\vert X_{1}^{(1)} \bigr\vert ^{s}\,\mathrm{d}x \\ &{}+\sum_{n=1}^{\infty}n^{r-2-q/p+s/2} \ln^{s} n \int_{n^{1/p}}^{\infty} x^{q-1-s}\bigl(\mathbb{E} \bigl(X_{1}^{(1)}\bigr)^{2}\bigr)^{s/2}\, \mathrm{d}x \\ \mathop{\hat{=}}& \tilde{J}^{(1)}_{1}+ \tilde{J}^{(1)}_{2}. \end{aligned}$$

If $u<2$, then by $\mathbb{E} |X|^{u}<\infty$ and $|X_{1}^{(1)}|\leq x^{\alpha}$,

3.9 $$\begin{aligned} \tilde{J}^{(1)}_{2} \leq&\sum_{n=1}^{\infty}n^{r-2-q/p+s/2}\ln^{s} n \int_{n^{1/p}}^{\infty} x^{q-1-s}\bigl(\mathbb{E} |X|^{u}\bigr)^{s/2}x^{\alpha(2-u)s/2}\,\mathrm{d}x \\ \ll&\sum_{n=1}^{\infty}n^{r-2-((\alpha u/p-1)/2+(1-\alpha)/p)s} \ln^{s} n< \infty \end{aligned}$$

from $q-1-(1-\alpha+\alpha u/2)s<-1$ and $r-2-((\alpha u/p-1)/2+(1-\alpha)/p)s<-1$.

If $u\geq2$, then $\mathbb{E}(X_{1}^{(1)})^{2}\leq\mathbb{E}X^{2}<\infty$. Hence,

3.10 $$ \tilde{J}^{(1)}_{2}\ll\sum_{n=1}^{\infty}n^{r-2-q/p+s/2}\ln^{s} n n^{(q-s)/p} =\sum _{n=1}^{\infty}n^{r-2-(1/p-1/2)s}\ln^{s} n< \infty $$

from $s>q$ and $r-2-(1/p-1/2)s<-1$.

For $\tilde{J}^{(1)}_{1}$, by the $C_{r}$ inequality and (2.1),

$$\begin{aligned} \tilde{J}^{(1)}_{1} \ll&\sum_{n=1}^{\infty}n^{r-1-q/p}\ln^{s} n \int_{n^{1/p}}^{\infty} x^{q-1-s} \bigl(x^{\alpha s}P \bigl(|X|>x^{\alpha}\bigr)+ \mathbb{E}|X|^{s}I\bigl(|X|\leq x^{\alpha}\bigr) \bigr)\,\mathrm{d}x \\ \leq&\sum_{n=1}^{\infty}n^{r-1-q/p} \ln^{s} n \int_{n^{1/p}}^{\infty} \bigl(x^{q-1-s+\alpha s-\alpha pr} \mathbb{E}|X|^{rp}+x^{q-1-s}\mathbb{E}|X|^{rp}x^{\alpha(s-rp)} \bigr)\,\mathrm{d}x \\ \ll& \sum_{n=1}^{\infty}n^{r-1-\alpha r-s(1-\alpha)/p} \ln^{s} n \\ < &\infty \end{aligned}$$

from $q-1-\alpha rp-s(1-\alpha)<-1$ and $r-1-\alpha r-s(1-\alpha)/p<-1$. By combining this with (3.3)-(3.5) and (3.7)-(3.10), we get that (3.2) holds. This ends the proof of (2.1) ⇒ (2.2).

Secondly we prove that (2.2) ⇒ (2.3). By (2.2) holds for any $\varepsilon>0$, we get

$$\begin{aligned}& \sum_{n=1}^{\infty}n^{r-2}\mathbb{E} \Bigl\{ \sup_{k\geq n}k^{-1/p}|S_{k}|-\varepsilon \Bigr\} _{+}^{q} \\& \quad =\sum_{j=1}^{\infty}\sum _{n=2^{j-1}}^{2^{j}-1} n^{r-2} \int_{0}^{\infty}P \Bigl(\sup_{k\geq n}k^{-1/p}|S_{k}|> \varepsilon+x^{1/q} \Bigr) \,\mathrm{d}x \\& \quad \ll\sum_{j=1}^{\infty}2^{(r-1)j} \int_{0}^{\infty}P \Bigl(\sup_{k\geq 2^{j-1} }k^{-1/p}|S_{k}|> \varepsilon+x^{1/q} \Bigr) \,\mathrm{d}x \\& \quad \ll\sum_{j=1}^{\infty}2^{(r-1)j} \sum_{i=j}^{\infty}\int_{0}^{\infty}P \Bigl(\max_{2^{i-1}\leq k< 2^{i} }k^{-1/p}|S_{k}|> \varepsilon+x^{1/q} \Bigr) \,\mathrm{d}x \\& \quad \ll\sum_{i=1}^{\infty}2^{(r-1)i} \int_{0}^{\infty}P \Bigl(\max_{2^{i-1}\leq k< 2^{i}}|S_{k}|> \bigl(\varepsilon+x^{1/q}\bigr)2^{(i-1)/p} \Bigr) \,\mathrm{d}x \\& \quad \ll\sum_{i=1}^{\infty}2^{(r-1-q/p)i} \int_{0}^{\infty}P \Bigl(\max_{1\leq k< 2^{i}}|S_{k}|> \varepsilon2^{(i-1)/p}+x^{1/q} \Bigr) \,\mathrm{d}x \\& \quad \ll\sum_{n=1}^{\infty}n^{r-2-q/p} \int_{0}^{\infty}P \Bigl(\max_{1\leq k\leq n}|S_{k}|> \varepsilon2^{-1/p}n^{1/p}+x^{1/q} \Bigr) \,\mathrm{d}x \\& \quad =\sum_{n=1}^{\infty}n^{r-2-q/p} \mathbb{E} \Bigl(\max_{1\leq k\leq n}|S_{k}|-\bigl( \varepsilon2^{-1/p}\bigr)n^{1/p} \Bigr)^{q}_{+} \\& \quad < \infty. \end{aligned}$$

That is, (2.3) holds.

Finally, we prove that (2.3) ⇒ (2.1). By (2.3) and $\max_{1\leq k\leq n}|X_{k}|\leq2\max_{1\leq k\leq n}|S_{k}|$, it follows that

$$\sum_{n=1}^{\infty}n^{r-2}\mathbb{E} \Bigl\{ \sup_{k\geq n}k^{-1/p}|X_{k}|-\varepsilon \Bigr\} _{+}^{q}< \infty \quad \mbox{for any } \varepsilon>0. $$

Therefore,

3.11 $$\begin{aligned}& \infty >\sum_{j=1}^{\infty}\sum _{n=2^{j-1}}^{2^{j}-1} n^{r-2}\mathbb{E} \Bigl(\sup _{k\geq 2^{j} }k^{-1/p}|X_{k}|-\varepsilon \Bigr)^{q}_{+} \\& \quad \gg\sum_{j=1}^{\infty}2^{(r-1)j} \mathbb{E} \Bigl(\max_{2^{j}\leq k< 2^{j+1}}2^{-(j+1)/p}|X_{k}|- \varepsilon \Bigr)^{q}_{+} \\& \quad \geq\sum_{j=1}^{\infty}2^{(r-1)j} \int_{0}^{\varepsilon^{q}}P \Bigl(\max_{2^{j}\leq k< 2^{j+1}}2^{-(j+1)/p}|X_{k}|> \varepsilon+x^{1/q} \Bigr) \,\mathrm{d}x \\& \quad \gg\sum_{j=1}^{\infty}2^{(r-1)j}P \Bigl(\max_{2^{j}\leq k< 2^{j+1}}|X_{k}|>2^{j/p}2^{1/p+1} \varepsilon \Bigr), \end{aligned}$$

it implies that $P(\max_{2^{j}\leq k< 2^{j+1}}|X_{k}|>2^{j/p}x)\rightarrow0$, $j\rightarrow\infty$ for any $x>0$. Thus, by Lemma 3.3, for any $x>0$, there is $c>0$ such that for sufficiently large *j*

$$2^{j}P\bigl(|X|>2^{j/p}x\bigr)\leq c P \Bigl(\max _{2^{j}\leq k< 2^{j+1}}|X_{k}|>2^{j/p}x \Bigr). $$

Consequently, taking $\varepsilon=2^{-1/p}$ in (3.11),

$$\begin{aligned} \infty >&\sum_{j=1}^{\infty}2^{(r-1)j} \int_{0}^{\infty}P \Bigl(\max_{2^{j}\leq k< 2^{j+1}}2^{-j/p}|X_{k}|-1>x^{1/q} \Bigr) \,\mathrm{d}x \\ \geq&\sum_{j=1}^{\infty}2^{rj} \int_{0}^{\infty}P \bigl(|X|>2^{j/p} \bigl(1+x^{1/q}\bigr) \bigr) \,\mathrm{d}x \\ =&\sum_{j=1}^{\infty}2^{(r-q/p)j} \int_{0}^{\infty}P \bigl(|X|>2^{j/p}+x^{1/q} \bigr) \,\mathrm{d}x \\ \geq&\sum_{j=1}^{\infty}2^{(r-q/p)j}\sum _{i=j}^{\infty}\int_{2^{iq/p}}^{2^{(i+1)q/p}} P \bigl(|X|>2^{j/p}+x^{1/q} \bigr) \,\mathrm{d}x \\ \gg&\sum_{j=1}^{\infty}2^{(r-q/p)j}\sum _{i=j}^{\infty}2^{iq/p} P \bigl(|X|>2^{i/p}\bigl(2^{1/p}+1\bigr) \bigr) \\ =&\sum_{i=1}^{\infty}\sum _{j=1}^{i}2^{(r-q/p)j} 2^{iq/p}P \bigl(|X|>c2^{i/p} \bigr) \\ \gg& \textstyle\begin{cases} \sum_{i=1}^{\infty}2^{ri}P (|X|>c2^{i/p} ),&\mbox{if }q< rp, \\ \sum_{i=1}^{\infty}2^{ri}\ln2^{i}P (|X|>c2^{i/p} ),&\mbox{if }q=rp, \\ \sum_{i=1}^{\infty}2^{iq/p}P (|X|>c2^{i/p} ),&\mbox{if }q>rp \end{cases}\displaystyle \\ \gg& \textstyle\begin{cases} \sum_{n=1}^{\infty}n^{r-1}P (|X|>cn^{1/p} )\gg\mathbb{E}|X|^{rp},&\mbox{if }q< rp, \\ \sum_{n=1}^{\infty}n^{r-1}\ln nP (|X|>cn^{1/p} )\gg\mathbb{E}|X|^{rp}\ln|X|,&\mbox{if }q=rp, \\ \sum_{n=1}^{\infty}n^{q/p-1}P (|X|>cn^{1/p} )\gg\mathbb{E}|X|^{q},&\mbox{if }q>rp. \end{cases}\displaystyle \end{aligned}$$

Hence, (2.1) holds. This completes the proof of Theorem 2.1. □

## References

[1] Chow Y (1988). On the rate of moment convergence of sample sums and extremes. Bull. Inst. Math. Acad. Sin..

[2] Li DL, Spătaur A (2005). Refinement of convergence rates for tail probabilities. J. Theor. Probab..

[3] Ebrahimi N, Ghosh M (1981). Multivariate negative dependence. Commun. Stat., Theory Methods.

[4] Lehmann EL (1966). Some concepts of dependence. Ann. Math. Stat..

[5] Joag-Dev K, Proschan F (1983). Negative association of random variables with applications. Ann. Stat..

[6] Liu L (2009). Precise large deviations for dependent random variables with heavy tails. Stat. Probab. Lett..

[7] Bozorgnia, A, Patterson, RF, Taylor, RL: Limit theorems for ND r.v.’s. Technical report, University of Georgia, Athens, Greece (1993)

[8] Wu QY, Jiang YY (2011). Strong consistency of *M* estimator in linear model for negatively dependent random samples. Commun. Stat., Theory Methods.

[9] Chen YQ, When AY, Ng KW (2010). The strong law of large numbers for extend negatively dependent random variables. J. Appl. Probab..

[10] Shen AT (2011). Probability inequalities for END sequence and their applications. J. Inequal. Appl..

[11] Shen AT (2016). Complete convergence for weighted sums of END random variables and its application to nonparametric regression models. J. Nonparametr. Stat..

[12] Wu YF, Song MZ, Wang CH (2014). Complete moment convergence and mean convergence for arrays of rowwise extended negatively dependent random variables. Sci. World J..

[13] Liu CC, Guo ML, Zhu DJ (2015). Equivalent conditions of complete convergence for weighted sums of sequences of extended negatively dependent random variables. Commun. Math. Res..

[14] Qiu DH, Chen PY (2015). Complete moment convergence for product sums of sequence of extended negatively dependent random variables. J. Inequal. Appl..

[15] Wang XJ, Zheng LL, Xu C, Hu SH (2015). Complete consistency for the estimator of nonparametric regression models based on extended negatively dependent errors. Statistics.

[16] Xu C, Xi MM, Wang XJ, Xia H (2016). $L_{r}$ convergence for weighted sums of extended negatively dependent random variables. J. Math. Inequal..

[17] Wang XH, Hu SH (2017). The strong consistency of *M*-estimates in linear models with extended negatively dependent errors. Commun. Stat., Theory Methods.

[18] Chen PY, Wang DC (2008). Convergence rates for probabilities of moderate deviations for moving average processes. Acta Math. Sin. Engl. Ser..

[19] Gut A, Stadtmüller U (2011). An intermediate Baum-Katz theorem. Stat. Probab. Lett..

[20] Sung SH (2013). Complete *q*th moment convergence for arrays of random variables. J. Inequal. Appl..

[21] Wang XJ, Hu SH (2014). Complete convergence and complete moment convergence for martingale difference sequence. Acta Math. Sin. Engl. Ser..

[22] Guo ML (2014). Equivalent conditions of complete moment convergence of weighted sums for *φ*-mixing sequence of random variables. Commun. Math. Res..

[23] Qiu DH (2014). Complete and complete moment convergence for weighted sums of widely orthant dependent random variables. Acta Math. Sin. Engl. Ser..

[24] Qiu DH, Chen PY (2014). Complete moment convergence for i.i.d. random variables. Stat. Probab. Lett..

[25] Wu QY, Jiang YY (2016). Complete convergence and complete moment convergence for negatively associated sequences of random variables. J. Inequal. Appl..

[26] Wu QY, Jiang YY (2016). Complete moment convergence for negatively dependent sequences of random variables. Discrete Dyn. Nat. Soc..

[27] Wu QY (2012). Sufficient and necessary conditions of complete convergence for weighted sums of PNQD random variables. J. Appl. Math..

